# Diversity of mosquito species and potential arbovirus transmission in long-tailed macaque (*Macaca fascicularis*) breeding facilities

**DOI:** 10.14202/vetworld.2022.1961-1968

**Published:** 2022-08-18

**Authors:** Dimas Novianto, Upik Kesumawati Hadi, Susi Soviana, Supriyono Supriyono, Lis Rosmanah, Huda Shalahudin Darusman

**Affiliations:** 1 Parasitology and Medical Entomology Laboratory, Animal Biomedicine Study Program, Graduate School, IPB University, Bogor, Indonesia; 2 Department of Animal Infectious Diseases and Veterinary Public Health, Faculty of Veterinary Medicine, IPB University, Bogor, Indonesia; 3 Primate Research Centre, Institute of Research and Community Service IPB University, Bogor, Indonesia; 4 Department of Anatomy, Physiology and Pharmacology, Faculty of Veterinary Medicine, IPB University, Bogor, Indonesia

**Keywords:** arbovirus, diversity, ecology, health, primates

## Abstract

**Background and Aim:**

Mosquito-borne viral infections are diseases that reduce human and animal health levels. Their transmission involves wildlife animals as reservoirs and amplifying hosts, including long-tailed macaques (*Macaca fascicularis*), and potentially transmits to humans and vice versa. This study aimed to determine the species diversity, richness, and biting activity of mosquitoes in a long-tailed macaque breeding area facility and discover the presence of *Flavivirus* and *Alphavirus* as the two main arboviruses reported to infect macaques.

**Materials and Methods:**

Human landing catch, light trap, and sweep net methods were used for mosquito collection around long-tailed macaques cages at parallel times for 12 h (18:00–06:00) for 12 nights. Mosquito species were identified to the species level based on the morphological identification key for Indonesian mosquitoes. Mosquito diversity was analyzed by several diversity indices. Mosquitoes caught using the human landing catch method were pooled based on mosquito species for viral ribonucleic acid extraction. Reverse transcription-polymerase chain reaction (RT-PCR) detected the non-structural protein 5 of the *Flavivirus* region and the non-structural protein 4 of the *Alphavirus* region. This study used the man-hour density and man-biting rate formulas for mosquito density.

**Results:**

Ten mosquito species were collected, namely, *Aedes albopictus*, *Anopheles aconitus*, *Anopheles minimus*, *Anopheles vagus*, *Armigeres foliatus*, *Armigeres subalbatus*, *Culex gelidus*, *Culex hutchinsoni*, *Culex tritaeniorhynchus*, and *Culex quinquefasciatus*. The number of mosquitoes caught using the light trap method had the highest abundance. In contrast, the number of mosquito species caught using the sweep net method had lower diversity than the other two methods. Seven mosquito species were obtained using the human landing catch method. The mosquito species with the highest density was *Cx. quinquefasciatus* within the observed densest period from 20:00 to 21:00. Negative results were obtained from RT-PCR testing on five species detected using universal *Flavivirus* and *Alphavirus* primers.

**Conclusion:**

The occurrence of mosquitoes in long-tailed macaque breeding facilities can be a source of transmission of zoonotic vector-borne diseases between animals and humans and vice versa.

## Introduction

In recent years, mosquito-borne viral infections, such as dengue virus (DENV; genus *Flavivirus*), chikungunya virus (CHIKV; genus *Alphavirus*), Japanese encephalitis virus (JEV; genus *Flavivirus*), and Zika virus (ZIKV; genus *Flavivirus*), have become a public health threat globally, including in Indonesia [1, 2]. The number of patients with arbovirus infections increases annually, and the number of mosquito vectors and the distribution of arboviruses in Indonesia also increase. In 2020, there were 138,127 dengue fever cases, with 747 deaths. The incidence rate of dengue fever was 40/100,000 population, with a fatality rate of 0.7%. In 2020, there were 1689 chikungunya fever cases reported in five Indonesian provinces [3]. In 2016, the Japanese encephalitis sentinel surveillance on children with acute encephalitis syndrome conducted in 11 Indonesian provinces revealed a Japanese encephalitis incidence of 15.2% [4]. ZIKV seropositivity in Indonesia was reported in children ages 1–4 years collected in 30 urban locations [5].

Most arboviruses circulate among wild animals. Many cause diseases after spillover transmission to humans, and domestic animals have acquired disease infections incidentally or sometimes become dead-end hosts [6]. The role of non-human primates as wild animals in spreading zoonotic mosquito-borne infections has been known in several studies. Serosurveillance in non-human primate-positive DENV and ZIKV is reported every 10 years (1960–2010) and summarized in several countries. In addition, several countries, including Malaysia and Senegal, have carried out DENV and ZIKV detection in the sylvatic cycle in primates [7]. Naturally, neotropical non-human primates can be infected by ZIKV [8]. Nakgoi *et al*. [9] detected DENV, JEV, and CHIKV antibodies in pig-tailed macaque colonies in captivity in North Thailand. Gutierrez-Bugallo *et al*. [10] conducted a metadata study on 31 wild-caught mosquito species infected with ZIKV. The total mosquito species were grouped into five genera, namely, *Aedes* (22 species), *Culex* (four species), *Anopheles* (two species), *Eretmapodites* (two species), and *Mansonia* (one species). *Stegomyia* is the major subgenus among *Aedes* mosquitoes from which ZIKV has been isolated (nine species). A serosurveillance of JEV in long-tailed macaques was recently reported in Bali and found 41.3% positivity for JEV antibodies [11].

*Macaca fascicularis* (long-tailed macaque) is a non-human primate widely distributed in Indonesia and is used as a research animal model in biology, biomedicine, and conservation by the Primate Research Center (PRC) of IPB University. The PRC of IPB University has a breeding facility for long-tailed macaques for various research purposes. Therefore, surveillance and monitoring activities for mosquito-borne viruses are needed to prevent arbovirus transmission in the breeding facility. Surveillance and monitoring activities can be initiated by knowing the diversity and abundance of mosquitoes in the breeding facility area.

This study aimed to determine the species diversity, richness, and biting activity of mosquitoes in a long-tailed macaque breeding area facility and discover the presence of *Flavivirus* and *Alphavirus* as the two main arboviruses reported to infect macaques.

## Materials and Methods

### Ethical approval

Ethical approval was obtained from the PRC Animal Ethics Committee, Institute for Research and Community Service, IPB University (animal ethical approval code IPB PRC-20-E005).

### Study period and area

The study was conducted from September 2020 to January 2021. The research was conducted in a long-tailed macaque breeding facility at the PRC of IPB University (6°33′22.0″S, 106°43′42.8″E), located at the IPB University Dramaga Campus, Bogor, West Java, Indonesia.

### Mosquito collection and identification

Mosquito collection was carried out for 12 nights with a collection time of 12 h (18:00–06:00). Human landing catch methods, light traps, and sweep nets were used to collect adult mosquitoes. The human landing catch method was carried out at night outside the long-tailed macaque breeding facility building area. The mosquito collector consisted of one person and caught mosquitoes for 50 min every 60 min for 12 h. The collector exposed his calves to knees as bait for mosquitoes to come to the collector, who then collected resting mosquitoes using an aspirator. Mosquitoes caught every hour were put into a labeled plastic cup. Mosquito collection using light traps was carried out parallel with the human landing catch. Four light traps were assembled on each outer side of the long-tailed macaque breeding facility building. A trap was assembled in the middle of the breeding facility building at the height of ±1.5 m above ground level. Mosquito collection using a sweep net was carried out around the breeding facility for 10 min every hour. The collected female mosquitoes were killed using chloroform and identified using *Aedes*, *Anopheles*, *Armigeres*, and *Culex* morphological key identification from Indonesia [12–15]. The collected male mosquitoes were not identified and excluded in this study.

### Viral ribonucleic acid (RNA) extraction and arbovirus detection from mosquito samples

Five mosquito species, consisting of one pool of *Aedes albopictus*, one pool of *Armigeres subalbatus*, one pool of *Culex hutchinsoni*, two pools of *Culex tritaeniorhynchus*, and five pools of *Culex quinquefasciatus*, were examined for arbovirus detection using polymerase chain reaction (PCR), following the procedure of Supriyono *et al*. [16] with PCR reagent modifications. RNA was extracted using tissue total RNA mini kit (Geneaid Biotech^®^, Taiwan), according to the manufacturer’s recommendations. The total volume of PCR was 25 μL, consisting of 2.5 μL RNA sample, 12.5 μL of 2× MyTaq One-Step Mix, 1 μL of each forward and reverse primer (10 pmol/μL), 0.25 μL RT enzyme, 0.5 μL RiboSafe RNase inhibitor, and 7.5 μL diethyl pyrocarbonate-H_2_O (Bioline, UK).

The primers used to amplify the sequences were MAMD and cFD2 with the non-structural protein target gene NS5 *Flavivirus* (252–260 bp long) and VIR2052F and VIR2052R with the non-structural protein target gene NS4 *Alphavirus* (144 bp long). Amplification was carried out using a SimpliAmp Thermal Cycler machine (Thermo Fisher Scientific, USA). The target gene, primer name, sequence, target size, and PCR conditions are presented in Table-1 [16]. The PCR product was electrophoresed on 1% agarose gel and visualized using an ultraviolet transilluminator.

**Table-1:** Target, primers, sequences, size, and cycle conditions used to detect *Flavivirus* and *Alphavirus* from mosquito pools collected from the long-tailed macaque breeding facility.

Gene target/region	Primer name	Sequence	Size (bp)	Cycle conditions	Reference
NS5 of the *Flavivirus* region	MAMD	5’-AACATGATGGGRAARAGRGARAA-3’	252–260	94°C, 2 min, 1 cycle 94°C, 1 min, 53°C, 1 min, 72°C, 1 min, 35 cycles 72°C, 5 min, 1 cycle	[16]
	cFD2	5’-GTGTCCCAGCCGGCGGTGTCATCAGC-3′			
NS4 of the *Alphavirus* region	VIR2052F	5’-TGGCGCTATGATGAAATCTGGAATGTT-3’	144	95°C, 15 min, 1 cycle 94°C, 30 s, 55°C, 30 s, and 72°C, 1 min, 35 cycles 72°C for 10 min	[16]
	VIR2052R	5’-TACGATGTTGTCGTCGCCGATGAA-3′			

### Statistical analysis

#### Mosquito diversity indices

Collected mosquito data were analyzed descriptively using several ecological indices consisting of the dominance number (D), Simpson diversity index (1-D), Shannon diversity index (H′), and Shannon–Weiner evenness (E). The Simpson diversity index (1-D) was used to measure species diversity for each collection method and was calculated as follows:


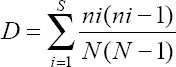


Where n is the total number of certain species (i) and N is the total number of individuals (in this case, the type of collection method). This index measured the probability that two specimens from one sample were of different species. The Simpson diversity index value increased along with the increase in diversity, and this index was sensitive to the abundance of the dominant species [17].

The Shannon diversity index was used to characterize species diversity in the three mosquito collection methods used in the study. The Shannon diversity index described the abundance and evenness of the species present. The study used a method of finding the pi value. The number of individuals of the *i* (ni) species was divided by the number of species (N) for the Shannon diversity index value. The pi value was multiplied by the natural logarithm of pi. The final results for each species were added and multiplied by −1 [17]. The Shannon–Weiner evenness value (E) was obtained by dividing the Shannon diversity index value by the natural logarithm of the total number of species from the collection method. The value of E ranged between 0 and 1, where 1 is complete evenness; that is, all species had the same abundance. The Shannon diversity index was calculated as follows:


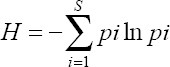


Dominance values and Shannon and Simpson indices were processed using the Paleontological Statistics Software Package for education and data analysis version 4.0 [18]. To visualize these values, a biodiversity profile was presented based on the Renyi index.

#### Density and biting activity

The man-hour density (MHD) and man-biting rate (MBR) formula was used to calculate the density of mosquitoes caught using the human landing catch method. The MHD and MBR values were analyzed descriptively to determine the anthropophilic species of mosquitoes and the density and activity of mosquitoes that suck blood. The MBR and MHD values were calculated as follows:


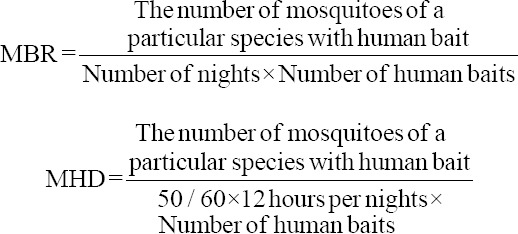


## Results

### Diversity and abundance of mosquitoes

**Figure-1:**
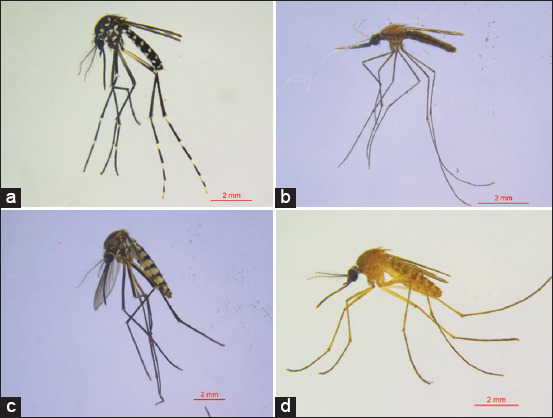
Four genera were caught in the long-tailed macaque breeding facility from September 2020 to January 2021: (a) *Aedes*, (b) *Anopheles*, (c) *Armigeres*, and (d) *Culex*.

There were 1108 female mosquitoes collected in the long-tailed macaque breeding facility, consisting of four genera: *Aedes*, *Anopheles*, *Armigeres*, and *Culex* (Figure-1). *Aedes*, *Armigeres*, and *Culex* genera were mosquitoes grouped into the Culicinae subfamily, whereas *Anopheles* was included in the Anophelinae subfamily. Anophelinae can be distinguished from other mosquitoes based on the morphological characteristics of the palpus and proboscis of the same length. Members of the subfamily Culicinae have shorter palpus than the proboscis. *Aedes* and *Culex* genera have shorter scales of wings, but the difference between both genera can be seen on the abdomen. *Aedes* has a pointed abdomen, whereas *Culex* has a blunt abdomen. *Armigeres* are morphologically larger than other Culicinae and usually have the proboscis slightly curved downward and flattened laterally.

The number of collected mosquito species by all collection methods used was 10 (Table-2). *Cx. quinquefasciatus* was the mosquito species with the highest abundance compared to other species, followed by *Ar. subalbatus* and *Ae. albopictus*. *Anopheles aconitus* and *Anopheles minimus* are the mosquitoes with the lowest abundance.

**Table-2:** Percentage of mosquito species caught using three different collection methods in the long-tailed macaque breeding facility from September to January 2021.

Mosquito species	Collection method			Total (%)

	Human landing catch (%)	Light trap (%)	Sweep net (%)	
*Ae. albopictus*	32 (28.9)	62 (5.60)	148 (13.36)	242 (21.85)
*An. aconitus*	1 (0.09)	0 (0)	0 (0)	1 (0.09)
*An. minimus*	0 (0)	1 (0.09)	0 (0)	1 (0.09)
*An. vagus*	0 (0)	28 (2.53)	0 (0)	28 (2.53)
*Ar. foliatus*	0 (0)	0 (0)	2 (0.18)	2 (0.18)
*Ar. subalbatus*	19 (1.71)	127 (11.46)	164 (14.80)	310 (27.97)
*Cx. gelidus*	2 (0.18)	0 (0)	0 (0)	2 (0.18)
*Cx. hutchinsoni*	3 (0.27)	11 (0.99)	0 (0)	14 (1.26)
*Cx. tritaeniorhynchus*	54 (4.87)	95 (8.57)	12 (1.08)	161 (14.52)
*Cx. quinquefasciatus*	128 (11.55)	151 (13.63)	68 (6.14)	347 (31.32)
Total	239 (21.56)	475 (42.87)	394 (35.56)	1108 (100)

*Ae. albopictus=Aedes albopictus, An. aconitus=Anopheles aconitus, An. minimus=Anopheles minimus, An. vagus=Anopheles vagus, Ar. foliatus=Armigeres foliatus, Ar. subalbatus=Armigeres subalbatus, Cx. gelidus=Culex gelidus, Cx. hutchinsoni=Culex hutchinsoni, Cx. tritaeniorhynchus=Culex tritaeniorhynchus, Cx. quinquefasciatus=Culex quinquefasciatus*

Table-3 presents the abundance, richness, diversity, evenness, and dominance of mosquitoes in the long-tailed macaque breeding facility. Seven mosquito species were caught using the human landing catch and light trap methods, whereas five were caught using the sweep net method. Four mosquito species were caught using the three methods, namely, *Ae. albopictus*, *Ar. subalbatus*, *Cx. tritaeniorhynchus*, and *Cx. quinquefasciatus*. The number of individuals caught using the light trap method was more than the other two methods.

**Table-3:** Abundance, richness, diversity, evenness, and dominance of mosquitoes in the long-tailed macaque breeding facility from September 2020 to January 2021.

Index	Collection method		

	Human landing catch	Light trap	Sweep net
No. of species (S)	7	7	5
No. of individuals (N)	239	475	394
Dominance (D)	0.362	0.223	0.345
Simpson (1-D)	0.637	0.766	0.654
Shannon (H)	1.259	1.572	1.169
Shannon–Weiner evenness (E)	0.50	0.69	0.64

**Figure-2:**
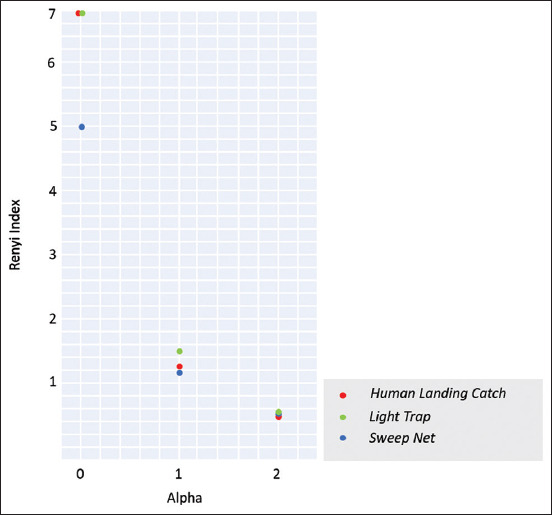
Diversity profile based on the Renyi index using three different collection methods in the long-tailed macaque breeding facility from September 2020 to January 2021.

The richness and diversity of mosquito species from the collection results using a diversity profile were based on the Renyi index (Figure-2). The zero-alpha value indicated the number of species found, the one-alpha value indicated Shannon’s diversity, and the two-alpha value indicated Simpson’s diversity. As presented in Table-3, the number of species and diversity of mosquitoes based on the Shannon index successfully collected using the human landing catch and light trap methods were higher than those collected using the sweep net method. However, the Simpson index value for mosquito collection using the sweep net method was higher than the human landing catch method. The Simpson diversity index value resulted from a reduction from one with a dominance number. If there were species that dominated, the diversity index value would be lower. In this study, the Shannon diversity index of mosquitoes in the long-tailed macaque breeding facility using human landing catch, light trap, and sweep net methods was 1.259, 1.572, and 1.169, respectively, which meant that the diversity values belonged to medium diversity. The Shannon–Weiner evenness value was 0.50, 0.69, and 0.64, respectively. The Shannon–Wiener evenness value was approaching 1, indicating that the collected mosquitoes were evenly distributed in captivity in the long-tailed macaque breeding facility by three different collection methods [19].

### Density and biting activity of mosquitoes and detection of arbovirus

**Figure-3:**
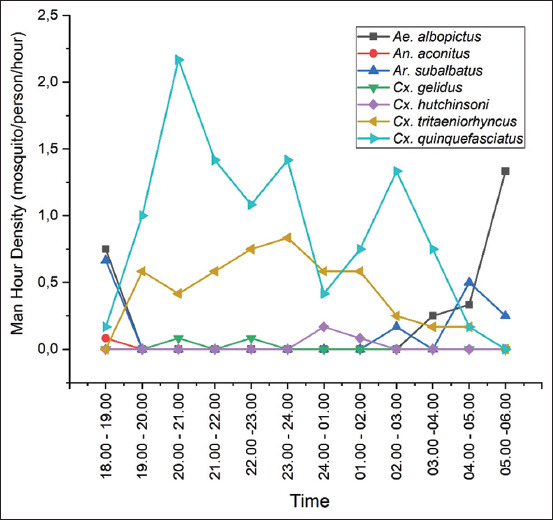
Man-hour density and biting activity of mosquitoes in the long-tailed macaque breeding facility from September 2020 to January 2021.

The mosquito density in the long-tailed macaque breeding facility was obtained by calculating the MHD and MBR values. Figure-3 shows the MHD value, which also describes the blood-biting activity during 12 h observation. Table-4 presents the MBR value.

**Table-4:** MBR of mosquitoes in long-tailed macaque breeding facility from September 2020 to January 2021.

Mosquito species	Density (mosquito/person/night)
*Ae. albopictus*	2.6
*An. aconitus*	0.27
*An. subalbatus*	1.58
*Cx. gelidus*	0.37
*Cx. hutchinsoni*	0.25
*Cx. tritaeniorhynchus*	4.5
*Cx. quinquefasciatus*	10.6

*Ae. albopictus=Aedes albopictus, An. aconitus=Anopheles aconitus, Ar. subalbatus: Armigeres subalbatus, Cx. gelidus=Culex gelidus, Cx. hutchinsoni=Culex hutchinsoni, Cx. tritaeniorhynchus=Culex tritaeniorhynchus, Cx. quinquefasciatus=Culex quinquefasciatus,* MBR=Man-biting rate

Results showed that mosquitoes biting human blood all night in the long-tailed macaque breeding facility had different activities. The biting behavior of *Cx. quinquefasciatus* at the study site occurred every hour of the night, with peak activity between 20:00 and 21:00 and increased again before and after midnight. Similar to *Cx. quinquefasciatus*, the biting activity of *Cx. tritaeniorhynchus* occurred throughout the night, with the highest activity between 23:00 and 00:00 and began to decline toward the morning. *Culex gelidus* bit the collector from 20:00 to 21:00 and 22:00 to 23:00, whereas *Cx. hutchinsoni* bit the collector from 12:00 to 01:00. This study also found that the biting activity of *Ae. albopictus* occurred in the early morning and at sunrise. Similar to *Ae. albopictus*, *Ae. subalbatus* performed biting activities from 18:00 to 19:00 and in the morning after 04:00.

The average MBR of 2.82 mosquitoes/person/night from 239 female mosquitoes was collected using the human landing catch method (Table-3). The three dominant mosquito species that came to the collector were *Cx. quinquefasciatus*, *Cx. tritaeniorhynchus*, and *Ae. albopictus*, with MBR values of 10.6, 4.5, and 2.6 mosquitoes/person/night, respectively.

**Figure-4:**
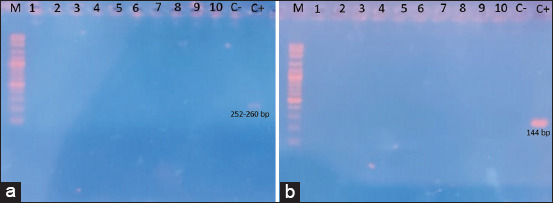
Electrophoresis results of polymerase chain reaction from 10 sample pools of mosquitoes caught using the human landing catch method: (a) *Flavivirus* and (b) *Alphavirus*: (1) *Aedes albopictus*, (2) *Armigeres subalbatus*, (3) *Culex hutchinsoni*, (4–5) *Culex tritaeniorhynchus*, and (6–10) *Culex quinquefasciatus*.

In the detection by reverse transcription-PCR, all samples tested in this study were negative for *Flavivirus* and *Alphavirus*. The results of electrophoresis of PCR products are presented in Figure-4.

## Discussion

Mosquito collection was carried out to determine the variety, abundance, and biting activity at the long-tailed macaque breeding facility. Non-human primates were reported to act as reservoirs for transmitting the mosquito-borne virus to human populations through competent mosquito vectors that feed on human and non-human primate blood [20]. This study was part of monitoring mosquitoes as vectors in the research location. The location was the long-tailed macaque breeding facility whose animals were used for biomedical research. The presence and abundance of mosquitoes at the research location can be a risk factor for disease transmission between employees or staff of PRCs and long-tailed macaques and vice versa through mosquito bites. Interestingly, the transmission of mosquito-borne pathogens between primates is that primates infected with pathogens clinically show no symptoms of illness but still experience viremia/parasitemia. Viremia/parasitemia conditions cause the virus/parasite to remain preserved in nature and can be transmitted through mosquito bites [21].

Three methods were used to collect mosquitoes in *M. fascicularis* breeding facility. Each collection method, along with other methods, aimed to complete the shortcomings of only one collection method. The limitations of this collection method were that animal baited traps were not used and the blood meal analysis was not conducted, so it is unknown which mosquito species have a preference to bite long-tailed macaque. The human landing catch method aimed to determine the anthropophilic mosquito species in *M. fascicularis* breeding facility [22]. The human landing catch method is the standard method for studying vector bionomics because this technique focuses on mosquitoes that feed on human blood, representing the most relevant proportion of the mosquito population for zoonotic mosquito-borne transmission [23].

Ten mosquito species were collected after 12 nights in *M. fascicularis* breeding facility. *Cx. quinquefasciatus* had the highest abundance of all species captured using three different methods. *Cx. quinquefasciatus* was reported as a competent vector of West Nile virus (WNV) in the Galapagos Islands and India [24, 25]. This mosquito species was reported to have a relative abundance of 13%. Based on a 2016 surveillance in Miami, Florida, WNV immunoglobulin G was found in *Macaca mulatta*, *M. fascicularis*, and *Papio hamadryas* [26]. Serological tests on mosquito salivary protein on *M. mulatta* and *Erythrocebus patas* found that *Cx. quinquefasciatus* had a bite preference for both non-human primates tested [27]. Uttah *et al*. [28] reported that the peak blood-sucking activity of *Cx. quinquefasciatus* in coastal areas of Nigeria occurred between 19:00 and 20:00. The blood-sucking activity of *Cx. tritaeniorhynchus* occurred throughout the night and had the highest activity between 23:00 and 00:00 and began to decline toward the morning.

*Ae. albopictus* was successfully collected with the second highest abundance. This species can be found in various environments throughout the tropics and subtropics. This species also inhabits both forested and peri-urban environments. Alencar *et al*. [29] revealed the natural infection and vertical transmission of ZIKV in *Ae. albopictus* originating from the sylvatic region. Host preference in *Ae. albopictus* can directly affect vector competence and the transmission risk of mosquito-borne disease pathogens. *Ae*. albopictus finds food in a hostile and opportunistic manner based on the environment and availability of a wide range of host dependents. When a choice is available, these species tend to bite humans (anthropophilic behavior), even if they can chase after the food from a large variant of animals, such as monkeys [30, 31].

The blood-biting behavior of *Ae. albopictus* has been widely studied in Asian countries. The blood-biting activity of this species occurs during the day (diurnal) and rarely at night. This species has a bimodal activity pattern, with one peak at sunrise and one in the afternoon [32]. This study found that *A. albopictus* bites in the early morning and at sunrise. Hadi *et al*. [33] reported the nocturnal activity of *Aedes aegypti* and *Ae. albopictus* also occurred in several areas in Indonesia. Similar to *Ae. albopictus*, *Ar. subalbatus* had biting behavior from 18:00 to 21:00 and in the morning after 04:00. This finding was in line with Pandian and Chandrasekan [34], who stated that the blood-biting behavior of *Ar. subalbatus* is crepuscular. It has two peaks of activity. The first peak activity occurs at dawn, and the second peak activity occurs in the afternoon, with the first peak activity being smaller than the second peak activity. Differences in peak activity were found between this and previous studies, namely, the peak of blood-sucking activity was higher in the afternoon than in the morning.

This study collected diurnal mosquitoes (*Ae. albopictus*), crepuscular mosquitoes (*Ar. foliatus* and *Ar. subalbatus*), and nocturnal mosquitoes (*An. aconitus*, *An. minimus*, *An.vagus*, *Cx. gelidus*, *Cx. hutchinsoni*, *Cx. tritaeniorhynchus*, and *Cx. quinquefasciatus*). Baik *et al*. [35] reported that nocturnal and diurnal mosquitoes have characteristics of light attraction and avoidance controlled by the circadian clock. They found physiological differences between circadian neural circuits and PER cycling in the central brain of nocturnal and diurnal mosquitoes. This underlined the differences in physiological changes and behavior of diurnal and nocturnal mosquitoes. Kawada *et al*. [36] suggested that the activity of looking for a host is positively correlated with an increase in light intensity. This information can be used as a basis for preventing mosquito bites not only during the day but also at night.

*Anopheles* was not obtained using the sweep net collection method. The most abundant *Anopheles* species is *An. vagus*, which was not observed going to the collector to suck blood. *An. vagus* is a zoophilic and exophilic mosquito, but this species plays an important role in malaria [37]. St. Laurent *et al*. [38] detected the sporozoite *Plasmodium vivax* in *An. vagus* using human and animal baits. *An. aconitus* was caught by one individual from 18.00 to 19.00. *An. vagus* and *An. aconitus* were confirmed to be the main vectors of malaria in several regions in Indonesia [39].

Arbovirus detection using two primers on five adult mosquito species that were successfully collected showed no *Flavivirus* and *Alphavirus* genetic material in the samples tested. This result indicated that the two viral genera tested did not circulate in long-tailed macaque breeding facilities. The negative results could also be due to the incorrect time of catching mosquitoes when the mosquito-borne virus in long-tailed macaque breeding facility or mosquitoes collected were nulliparous.

The shortage of this research is the lack of virus isolation derived from mosquito samples. Determination of arbovirus infection status in long-tailed macaques can be done by isolating the virus in long-tailed macaques and measuring the relatively high prevalence of antibodies in long-tailed macaques. Transmission arbovirus in long-tailed macaque populations must have a sufficient population of infected mosquitoes simultaneously spatially and temporarily competent (capable of transmitting the virus) to bite long-tailed macaques. Activities to describe arbovirus transmission in long-tailed macaques or other non-human primates include collection and identification of mosquitoes, detection of viruses in mosquitoes, blood meal analysis, observation of mosquitoes that feed on long-tailed macaque blood, and experiments on the transmission of arbovirus between mosquitoes and long-tailed macaques in the laboratory [7].

## Conclusion

A long-tailed macaque breeding facility is a potential site for arbovirus circulation because it supplies a blood source for mosquitoes in the vicinity of the breeding facility. Undetectability of target virus genes in the tested mosquitoes should not reduce the awareness of zoonotic mosquito-borne transmission with non-human primate reservoirs because the mosquito species collected are competent vectors of various pathogens. Population reduction and mosquito elimination are methods to reduce or prevent the malaria transmission risk. Activities include eliminating the potential breeding sites around the breeding facility and closing the gaps in the semi-open breeding facility building to prevent mosquitoes from sucking the blood of long-tailed macaques as arbovirus reservoir hosts.

## Authors’ Contributions

UKH, SS, SU, LR, and HSD: Designed and supervised the study and revised the manuscript. DN: Sample collection. DN, UKH, SS, SU, LR, and HSD: Laboratory works and drafted the manuscript. All authors have read and approved the final manuscript.
